# Axillary enlargement in gestational gigantomastia, an uncommon presentation of a rare disease. A case report

**DOI:** 10.1016/j.ijscr.2020.05.001

**Published:** 2020-05-19

**Authors:** Mohammad Naser Athamnah, Mohammad Saleh Al-Share, Enas Mohammed Hajjaj, Liqa N. Athamneh, Omar M. Abuelaish

**Affiliations:** a Jordan Medical Council, Jordan; b San Gerardo Hospital, Milano, Italy; c Jordan Ministry of Health, Jordan; d Department of Family Medicine, Jordan Ministry of Health, Jordan; e Translational Biology Medicine and Health - Virginia Tech, USA; f Jordan Royal Medical Services, Jordan

**Keywords:** Gestational gigantomastia, Gravid macromastia, Axillary enlargement, Conservative management, Case report

## Abstract

**Background:**

Gestational gigantomastia presents in pregnancy as a rapid and excessive enlargement of the breast reducing the quality of life and causing psychological and physical impairment. Huge axillary enlargement due to Gestational gigantomastia is not mentioned in the literature before. Best treatment remains unclear as conservative and medical management are options beside the more aggressive surgical treatment.

**Case presentation:**

This is a special case of Gestational gigantomastia presented by huge axillary and breast enlargement in a 34-year-old female. Treated successfully in a conservative manner, a near complete spontaneous resolution happened two months post-delivery.

**Conclusion:**

Axillary involvement is not mentioned in the limited literature discussing the rare disease of gestational gigantomastia. Not only breast but also axillary area can be involved and enlarge due to this disease. Conservative non-operative management was successful.

## Highlights

• Gestational gigantomastia presents in early pregnancy as huge continuous breast size enlargement.
• Axillary involvement is not mentioned in medical literature before.
• Conservative management of Gestational gigantomastia is a valid option depending on patient condition and tolerance.

## Graphical abstract

**Figure:**
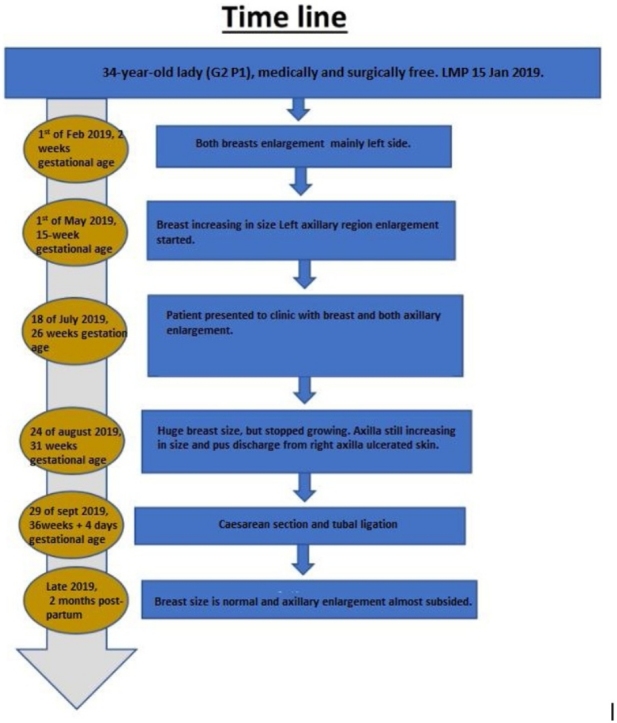

## 1 Introduction

Having an unknown aetiology, Gestational gigantomastia (i.e. Gravid macromastia) presents in pregnancy as a rapid and excessive enlargement of the breast reducing the quality of life and causing psychological and physical impairment. Severe discomfort and back pain, also skin necrosis and serious infections are all possible complications for this rare disease that is affecting 1 in every 100.000 pregnancies [1,2]. Breast enlargement is the main presentation of Gestational gigantomastia in cases reported in medical literature [3]. Significant axillary enlargement is not mentioned. Management of Gestational gigantomastia can be conservative, medical or surgical [3]. This case report is showing that axillary enlargement can be part of the presentation of Gestational gigantomastia, in the same time supporting the effectiveness of conservative management for Gestational gigantomastia. This case report is following the SCARE criteria for case reports publication [4].

## 2 Case report

This is a 34-year-old lady (G2 P1), medically and surgically free. History of mild breast enlargement during the first pregnancy (3 years ago) which subsided spontaneously after delivery.

Early February 2019, patient was 2 weeks pregnant and started to complain of breast discomfort with mild enlargement mainly on the left side. Breast discomfort and enlargement continued to increase for the coming months. By may 2019, patient started to notice and feel the axillary enlargement in both sides specially the left one. Mid July 2019, at 26 weeks gestation age, patient was referred to us from the OB/GYN for a surgical consultation regarding her breast problem.

**Fig. 1:**
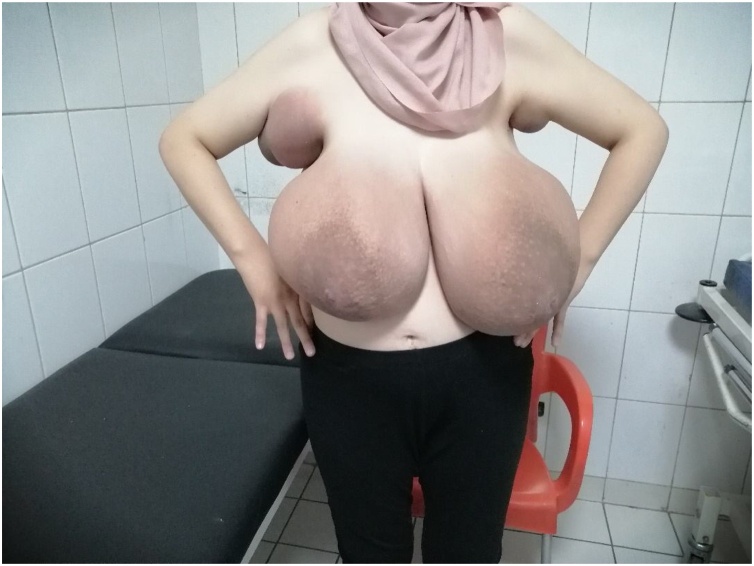
Patient presenting with breast and bilateral axillary enlargement.

**Fig. 2:**
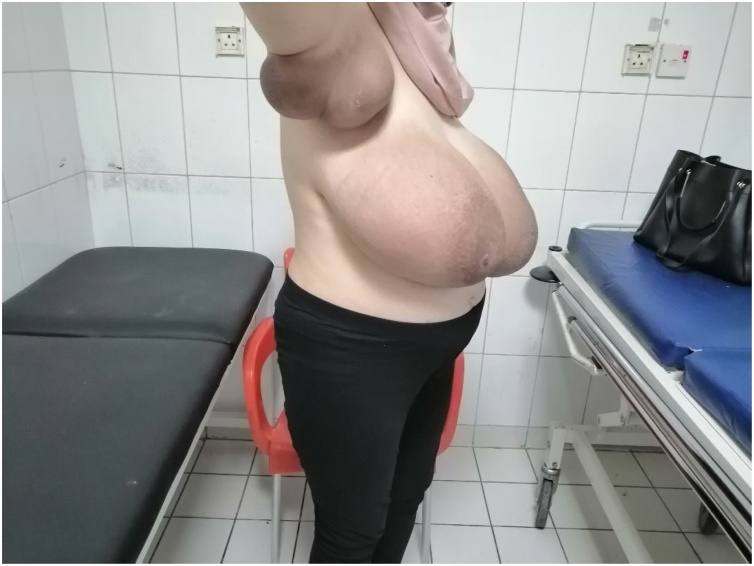
Enlarged right axilla at 26 gestational weeks.

Upon physical exam, healthy 26 weeks pregnant woman. Breast exam revealed a massive enlargement in both breasts and the axillary region bilaterally mainly right side. Skin redness with non-significant ulceration on both breasts, dilated superficial veins and signs of skin stretch. Both axillary areas were enlarged and tender with apparent red discoloration, Fig. 1. Physical exam revealed no isolated breast masses rather than global enlargement and axillary involvement. Fig. 2.

**Fig. 3:**
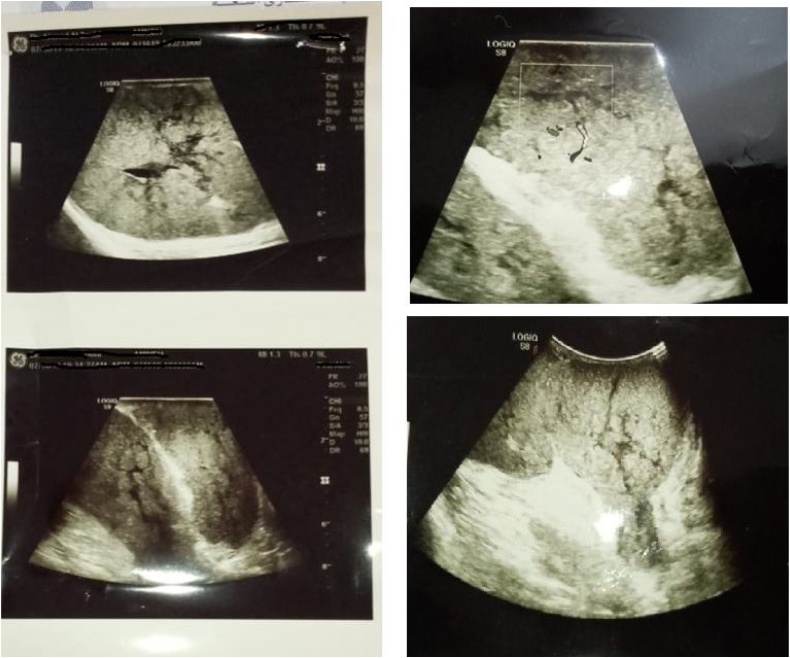
Hypervascular huge axillary lymphatic enlargement and bilateral accessory breast.

Complete blood count, Liver function tests, Kidney function test, complete hormonal profile tests were ordered by OB/GYN specialist and showed normal results. Breast mammography was avoided, breast ultrasound showed no definite breast masses other than the global hypertrophy and enlargement, axillary ultrasound suggested the presence of bilateral accessory breast and hypervascular huge lymphatic enlargement, Fig. 3. Patient diagnosed as Gestational gigantomastia with axillary enlargement.

Treatment plan was based on conservative management as the patient reported self-limiting breast enlargement in her previous pregnancy, although considerably less than this time and without axillary involvement. Patient was advised to wear a supporting bra and local care creams for her breast and axillary skin was prescribed. Close follow up and monthly appointments were scheduled for her.

**Fig. 4:**
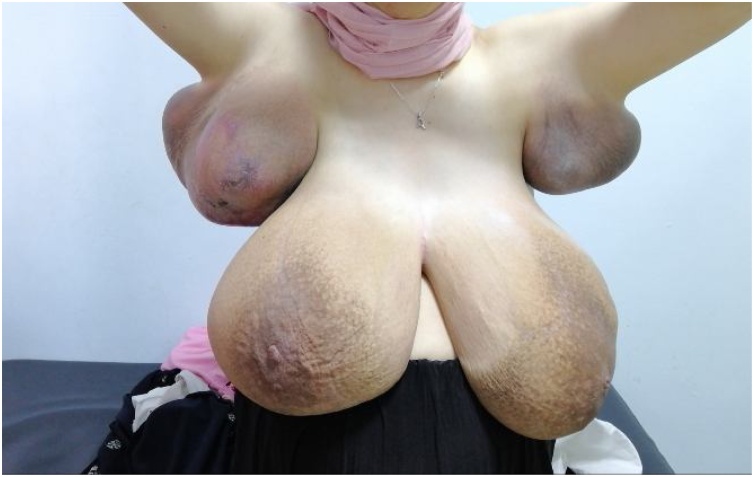
Breast and bilateral axillary involvement at 31 gestational weeks.

**Fig. 5:**
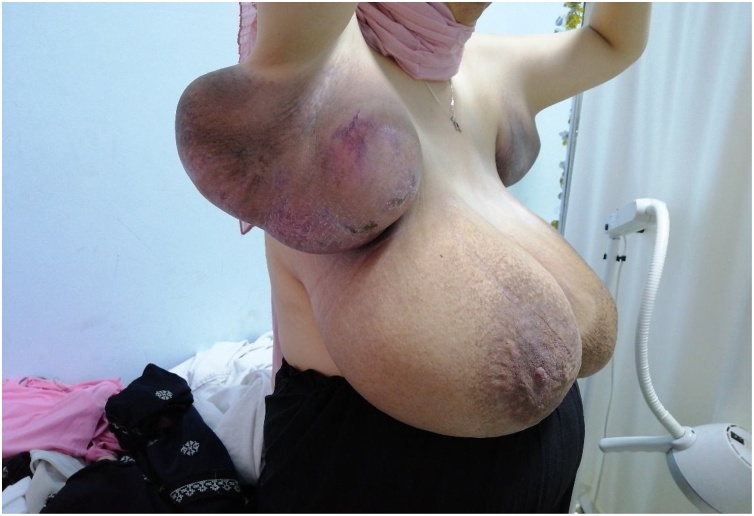
Massive axillary enlargement with ulceration at 31 gestational weeks.

By the 24th of august 2019 at 31 weeks gestational age, she presented with stable enlarged breast size. But, axillary exam revealed an increasing in size compared with last visit and minimal pus discharge from right axilla ulcerated skin, Fig. 4, Fig. 5. Penicillin based anti-biotic was prescribed with proper local control. Mid-September, she presented again with right axillary skin ulceration, but no pus discharge. At this point Breast and axillary size were massive, but stable.

**Fig. 6:**
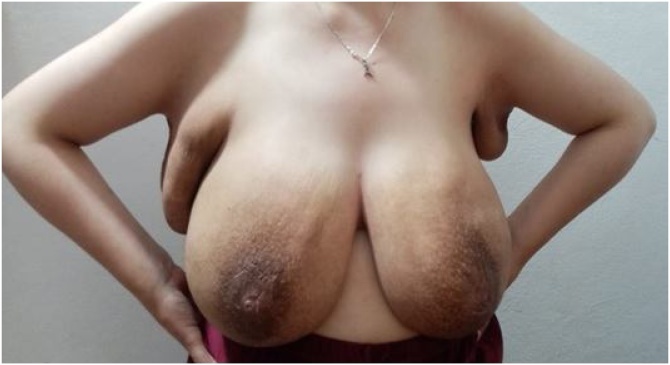
Breast enlargement subsided, axillary lymph nodes decreased in size.

Electively, on the 29 of sept 2019, at 36weeks + 4 days gestational age a caesarean section with bilateral tubal ligation was performed, delivering a 3 kg healthy baby. Ten days post-partum a gradual decrease in breast and axillary size started to take place and by the end of 2019, 2 months post-partum, breast size is normal and axillary enlargement and engorgement almost subsided, Fig. 6.

## 3 Discussion

Gestational gigantomastia usually presents as massive breast enlargement during the first trimester, (Mangla & Singla, 2017). As Gestational gigantomastia is a rare disease with uncertain aetiology, cases are being treated on individual bases [5], management of Gestational gigantomastia is not standardised and ranges from conservative to medical and surgical treatment [3,5]. Treatment is expected to be individualized according to the specific case and severity of symptoms. Symptoms that are significantly affecting one patient and limiting her daily activities might be tolerated by another patient.

Very few cases are reported as spontaneous resolution post-partum [6,7]. If breast enlargement is tolerated by the patient and not a life-threatening condition, it is worth trying conservative and supportive measurements such as a supporting bra and proper skin hygiene [5].

In cases of symptoms such as sever back pain, shortness of breath, breast pain or skin ulceration and infection, initiation of medical management to reduce breast size might be necessary [7]. Medical treatment is mainly by Bromocriptine, a dopamine agonist. Although its safety is proven during pregnancy, but intra-uterine growth retardation cases were reported [3,5]. Abortion and congenital malformations are not reported to be higher in women taking Bromocriptine [3]. Medical management is limited as only few cases of Gestational gigantomastia responded to Bromocriptine [3,5,8].

Whenever the symptoms of the disease are not tolerated by the patient and not life threatening, a trail of medical management is warranted, if not successful and disease remain in progress or patient develop serious complications, then surgical management is justified, surgical management with mastectomy or breast reductions is reserved as a last choice and should only be performed in cases of sever disease and significant morbidity [8].

Regarding patient in this case report, the decision of conservative management for Gestational giganomastia was guided by her history of the self-limiting similar complain in her previous pregnancy and the published case reports describing spontaneous resolution [6,7]. After proper counselling and conversation with our patient we reached the decision of conservative management.

Surgery was not discussed, as she tolerated symptoms very well and she experienced similar condition in her first pregnancy, with complete resolution after delivery. Bromocriptine medication was advised, but fear of possible side effects during pregnancy, e.g. intrauterine growth retardation was weighed and patient decided to choose conservative management after all.

The treatment of right axillary infection was based on the safety of Penicillin in pregnancy and susceptibility of such skin infections to this antibiotic [9].

After the patient was discharged home with her baby, in her follow up visit she reported her satisfaction as no surgical operations were performed and mastectomy was avoided, a mastectomy would have left her with significant psychological distress.

Understanding the disease and reaching a standardized efficient treatment plan is limited by the small number of cases diagnosed and reported, reflecting how infrequent this disease is. In current available literature, most cases are treated surgically, few are successfully conservatively or medically managed [8].

Reporting, documentation and publishing of all cases of Gravid macromastia and their course of treatment, complications and long term follow up is needed. This will allow more systematic literature reviews and meta-analysis studies that helps in creating guidelines and standardizing the treatment.

As in our case, the multi-disciplinary team including breast surgeon, OB/GYN and paediatrics is necessary. Induction of early delivery, the need for caesarean section and risk of pre-term baby requiring extra care by neonatologist are all possible scenarios mandating a multi-disciplinary team. It was the patient choice to undergo bilateral tubal ligation to prevent future pregnancy as the patient and her husband are satisfied with two healthy daughters.

The case presented here is adding to the literature the presence of significant axillary enlargement in the scenario of Gestational gigantomastia, in the same time supporting the effectiveness of conservative management for Gestational gigantomastia. Almost complete spontaneous resolution of the breast disease and massive reduction in axilla size was noticed after delivery. Avoidance of any invasive methods to treat Gestational gigantomastia was possible in this patient.

## 4 Conclusion

Gestational Gigantomastia is a rare disease with limited literature about this topic. Axillary enlargement was unique to our patient among similar case reports. Conservative treatment leading to spontaneous resolution of symptoms after delivery was the course of our patient. Aggressive surgical management with mastectomy or reduction mammoplasty should be reserved for sever cases or cases with no resolution after delivery.

## Declaration of Competing Interest

No conflicts of interest.

## Funding

No sources of funding.

## Ethical approval

No ethical approval is needed for case reports as long as we have written patient consent and patient is anonymised.

## Consent

I have written consent signed by the patient.

Printed and signed in patient native language: Arabic.

## Author contribution

Mohammad Naser Athamnah: Conceptualization, Investigation, Writing - Original Draft, Writing - Review & Editing, Visualization and Project administration.

Mohammad Saleh Al-Share: Writing - Original Draft and Supervision.

Enas Mohammed Hajjaj: Patient care, follow up and Data collection.

Liqa N. Athamneh: Review & Editing.

Omar M. Abuelaish: Writing - Draft.

## Registration of research studies

NA.

## Guarantor

Mohammad Naser Athamnah.

## Provenance and peer review

Not commissioned, externally peer reviewed.
